# Prognostic impact of complete metastasectomy in metastatic renal cell carcinoma in the era of immuno-oncology-based combination therapies

**DOI:** 10.1007/s00345-022-03960-1

**Published:** 2022-02-26

**Authors:** Viktoria Stühler, Lisa Herrmann, Moritz Maas, Simon Walz, Steffen Rausch, Arnulf Stenzl, Jens Bedke

**Affiliations:** Department of Urology, University Hospital Tuebingen, Eberhard-Karls-University Tuebingen, Tübingen, Germany

**Keywords:** Immuno-oncology, Metastasectomy, Renal cell carcinoma, Tyrosine kinase inhibitor

## Abstract

**Purpose:**

Complete metastasectomy of renal cell carcinoma (RCC) is receding into the past due to the progress of immuno-oncology-based combinations (IO) in systemic therapy. The prognostic impact of curative intended complete metastasectomy vs. immediate IO-based therapy or tyrosine kinase inhibition (TKI) on progression-free survival (PFS) and cancer-specific survival (CSS) was investigated in the first-line setting.

**Methods:**

205 patients with synchronous or metachronous metastasis received complete metastasectomy (*n* = 80) or systemic therapy (*n* = 125, TKI: 87, TKI–IO: 13, IO–IO: 25) as first-line therapy. The prognostic impact of these therapies was assessed using Cox regression and Kaplan–Meier analyses.

**Results:**

First-line complete metastasectomy significantly improved CSS compared to both TKI monotherapy (6.1 vs. 2.6 years, HR 0.45, *p* < 0.001) and IO-based combination therapy (IO–IO/TKI–IO, 6.1 vs. 3.5 years, HR 0.28, *p* = 0.007). Repetitive complete metastasectomy without ever receiving systemic therapy vs. systemic therapy in first-line significantly prolonged CSS (11.3 vs. 3.1 years, HR 0.34, *p* = 0.002). First-line complete metastasectomy and subsequent systemic therapy at tumor progression was associated with a significant CSS benefit vs. systemic therapy (5.8 vs. 3.1 years, HR 0.53, *p* = 0.003), also compared to IO-based combinations (5.8 vs. 3.5 years, HR 0.30, *p* = 0.017). Median PFS was improved by IO-based therapy compared to TKI monotherapy in the first-line setting (HR 0.61, *p* = 0.05), with maximal benefit of the TKI–IO combination vs. TKI monotherapy (HR 0.27, *p* = 0.01), as well as compared to PFS of complete metastasectomy (HR 0.34, *p* = 0.035).

**Conclusion:**

Despite the progress of IO-based combination therapies in first line, complete metastasectomy remains an integral part of the multimodality treatment of metastatic RCC.

## Introduction

Approximately 20–30% of patients with newly diagnosed renal cell carcinoma (RCC) have synchronous metastasis at initial diagnosis, and for localized tumors, up to 40% of patients develop metastases after nephrectomy during follow-up depending on individual risk factors [1, 2]. With better understanding of the genetic and molecular mechanism of RCC tumorigenesis, tyrosine kinase inhibitors (TKIs) and immuno-oncology-based drugs (IO) have emerged and offer survival benefits in some patients with advanced disease [3]. In accordance with the current guidelines of the European Association of Urology (EAU), metastasectomy is recommended for patients in whom complete surgical resection is technically feasible or even for local control of symptoms [4]. Previous studies have shown that complete surgical resection of RCC metastases is associated with a survival benefit [5–8]. Nevertheless, metastasectomy remains one of the options to achieve a complete and potentially durable cure in selected patients with or without systemic therapies. Indeed, most of the data supporting this strategy come from the past cytokine and TKI era. The role of complete metastasectomy in the IO-based era is not well studied. We therefore aimed to investigate the survival of patients with metastatic RCC (mRCC) with complete metastasectomy in the era of targeted therapies and checkpoint inhibitors in the current study.

## Material and methods

205 patients with synchronous or metachronous mRCC received curative intended complete metastasectomy (*n* = 80) or systemic therapy (*n* = 125) with TKI monotherapy (*n* = 87) or an IO-based therapy (*n* = 28) as first-line therapy at the Department of Urology of the University of Tuebingen, Tuebingen, Germany. The prognostic impact of these therapies was examined using Cox regression and Kaplan–Meier analyses.

Clinical data collected included time of primary surgery, TNM stage, grading, histological subtype, and ECOG performance status. Index metastatic characteristics included timing of nephrectomy, number of distinct metastases, metastatic site, first-line, and subsequent therapies. In addition, the Memorial Sloan-Kettering Cancer Center (Motzer, MSKCC) risk score was calculated at the time of first metastasis. Complete metastasectomy was defined as complete resection of all index sites of metastasis. Progression-free survival (PFS) and cancer-specific survival (CSS) rates were estimated using the Kaplan–Meier method, with follow-up time calculated from the date of index metastasis. Associations with time to death from RCC were assessed using Cox proportional hazards regression models and summarized with hazard ratios (HR) and 95% confidence intervals (CI). For multivariate analysis, clinically important parameters were included in addition to those significant in univariate analyses. The MSKCC score was chosen instead of the single variables performance status and time from nephrectomy to metastasis, as it summarizes the most important clinical parameters. Statistical analyses were performed using SPSS, version 27. A *p* < 0.05 was considered statistically significant.

## Results

**Fig. 1:**
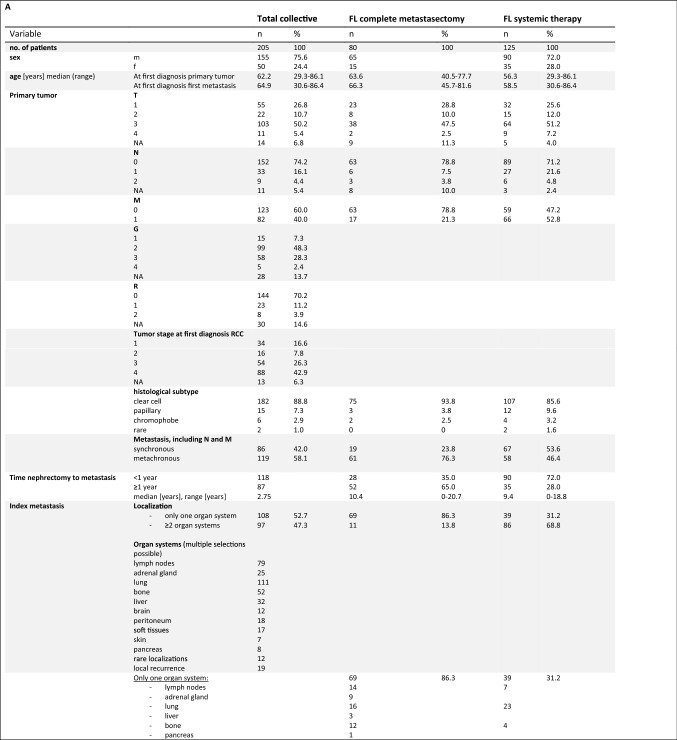

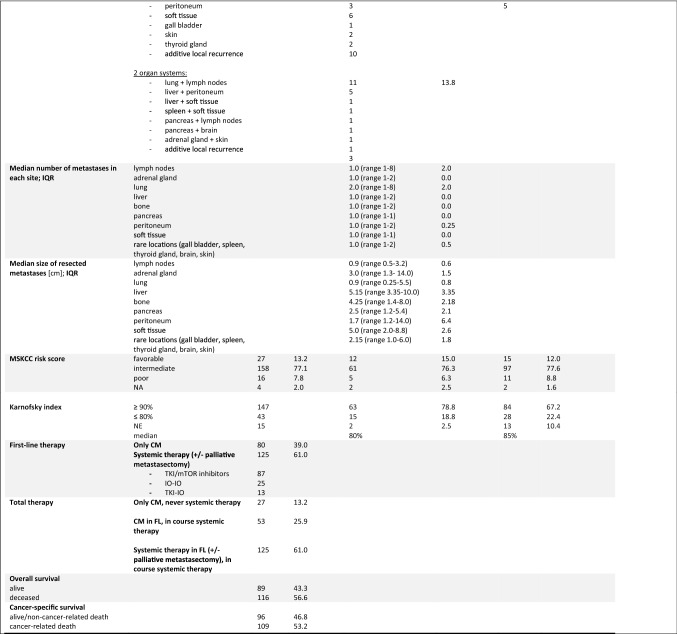

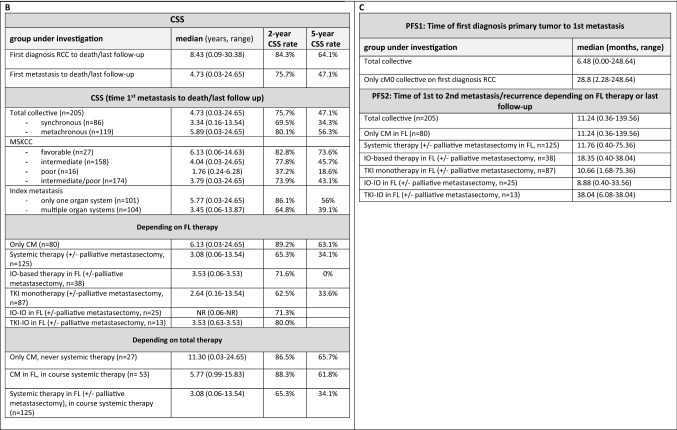
**A** Baseline characteristic of the total study cohort and in the subgroup of patients with first-line complete metastasectomy or first-line systemic therapy. **B** Overview of calculated CSS depending on clinical parameters as well as first-line therapy and overall therapy.** C** A tabulated summary of calculated PFS as a function of first-line therapy. *CM* complete metastasectomy, *CSS* cancer-specific survival, *FL* first-line therapy, *G* grading, *IQR* interquartile range, *IO* immuno-oncology, *M* distant metastasis, *MSKCC* Memorial Sloan-Kettering Cancer Center (Motzer) Score, *N* regional lymph nodes, *NE* not evaluable, *PFS* progression-free survival, *R* resection status, *T* primary tumor, *TKI* tyrosine kinase inhibitor

Among 205 RCC patients with synchronous (*n* = 86) or metachronous (*n* = 119) metastasis, 80 patients underwent curative intended complete metastasectomy and 125 patients received primary systemic therapy, with 87 patients treated with a TKI monotherapy, 13 with a TKI–IO combination, and 25 with an IO-monotherapy or IO–IO combination as first line. Ipilimumab plus nivolumab (*n* = 23) or pembrolizumab monotherapy (*n* = 2) were the agents used in the later mentioned group, while various combinations with axitinib plus pembrolizumab (*n* = 6), lenvatinib plus pembrolizumab (*n* = 1), and cabozantinib plus IO (*n* = 6) were given for the TKI–IO combination. The median follow-up from the time of first metastasis was 2.61 years (range 0.03–24.65 years), 116 patients died, including 109 of RCC. Baseline characteristics of the total study cohort and the subgroups of patients with first-line complete metastasectomy or first-line systemic therapy are shown in Fig. 1A. Regarding the 119 clinical cases without metastasis at nephrectomy (cM0), the median time to index metastasis was 2.4 years (range 0.19–20.72 years). Overall, synchronous compared to metachronous metastasis was a poor prognostic factor in terms of CSS (HR 1.61, *p* = 0.014). Interestingly, MSKCC prognostic score was not a significant prognostic factor (*p* = 0.091), whereas CSS was significantly improved for patients with only evidence of metastases in one organ system as compared to those with multiple organ systems affected at initial diagnosis of metastasis (HR 0.60, *p* = 0.009).

**Fig. 2:**
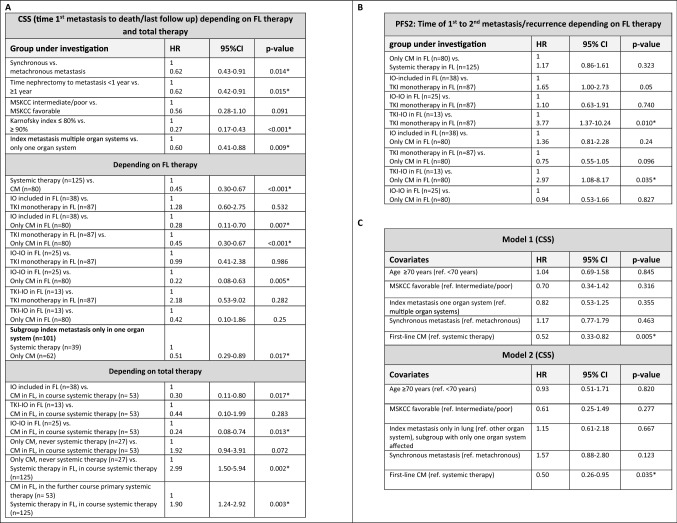

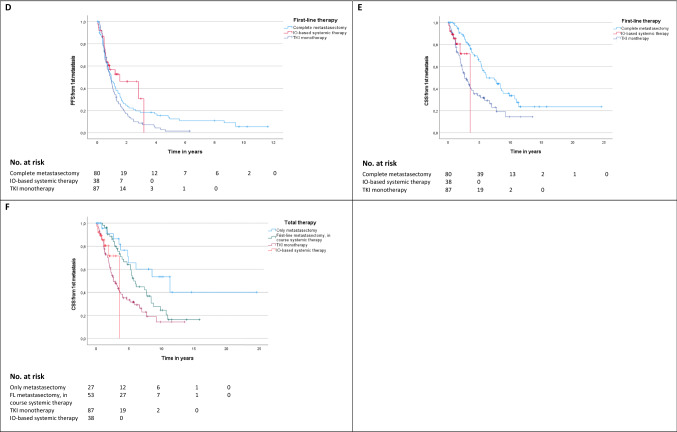
**A** Univariate analysis of CSS, defined as time from first metastasis to death/last follow-up, depending on first-line and overall therapy. First-line therapy with systemic therapy always includes the patient group with or without palliative metastasectomy. **B** Univariate analyses of PFS2, defined as time from 1st to 2nd metastasis/relapse, derived from first-line therapy. First-line therapy with systemic therapy here always includes the patient group with or without palliative metastasectomy. **C** Multivariate analyses of CSS for clinical parameters. Kaplan–Meier analyses for PFS depending on first-line therapy **(D)**, for CSS depending on first-line therapy **(E)**, and for CSS depending on total therapy **(F).** *CM* complete metastasectomy, *CSS* cancer-specific survival, *FL* first-line therapy, *IO* immuno-oncology, *M* distant metastasis, *MSKCC* Memorial Sloan-Kettering Cancer Center (Motzer) score, *PFS* progression-free survival, *Ref*. reference, *TKI* tyrosine kinase inhibitor

Depending on first-line therapy, median CSS was longest for patients treated with curative intended complete metastasectomy with 6.1 years (range 0.36 months–24.65 years) with a 2- and 5-year CSS rates of 89.2% and 63.1%. Thereby, CSS was significantly improved in patients with curative first-line complete metastasectomy compared to systemic therapy in general (median CSS: 6.1 vs. 3.1 years, HR 0.45, *p* < 0.001) as well as to TKI monotherapy (median CSS: 6.1 vs. 2.6 years, HR 0.45, *p* < 0.001), but this survival benefit was also present when patients were treated with an IO-based combination (IO–IO/TKI–IO, median CSS 6.1 vs. 3.5 years, HR 0.28, *p* = 0.007). The corresponding Kaplan–Meier curves are shown in Fig. 2E. However, when subdividing the IO-based group, the subgroup of patients with first-line IO–IO combination showed a significant worsening of CSS compared to complete metastasectomy (HR 4.55, *p* = 0.005), while for first-line TKI–IO combination the median CSS was improved at 6.1 years, although the difference compared to complete metastasectomy did not reach statistical significance (HR 2.38, *p* = 0.25). Interestingly, there was no difference on CSS for first-line IO-based therapy compared to TKI monotherapy (*p* = 0.532). This was also confirmed for the subgroups of first-line IO–IO combination compared to TKI monotherapy (HR 1.01, *p* = 0.986), while CSS comparisons for the TKI–IO combination vs. TKI monotherapy did not reach the threshold of statistical significance, even though HR and 95% CI suggested the potential for a significant association. Although our study did not have sufficient power to demonstrate this (HR 0.46, *p* = 0.282). A subgroup analysis including patients with index metastasis in only one organ system confirmed the benefit of first-line complete metastasectomy compared to systemic therapy (HR 0.51, *p* = 0.017). Multivariate analysis confirmed first-line complete metastasectomy as an independent prognosticator to be significantly associated with better CSS (see models 1 and 2 in Fig. 2C).

Considering the overall treatment course, median CSS was by far the highest at 11.3 years (range 0.03–24.65 years) for patients single or repetitive complete metastasectomy without ever having received systemic therapy. For this group, the median number of complete metastasectomies was 1 with a range between 1 and 7. Metastasis subsequently developed in 67 of the 80 patients (83.8%) treated with complete metastasectomy in first line. Median subsequent metastasis-free survival following complete metastasectomy in first line was 9.2 months (range 0.72 months–9.4 years). This was followed by patients with first-line complete metastasectomy and subsequent systemic therapy at tumor progression with a median CSS of 5.77 years (range 11.9 months–15.83 years). The worst median CSS was 3.08 years (range 0.7 months–13.54 years) among the subgroup of patients treated with systemic therapy from first line, with 2- and 5-year CSS rates of 65.3% and 34.1%, respectively. Once more, the appropriate Kaplan–Meier curves for CSS depending on the total therapy are given in Fig. 2F. Furthermore, Fig. 1B provides an overview of the calculated CSS depending on clinical parameters as well as first-line and overall therapy.

Here, in univariate analyses, the median CSS was significantly prolonged for patients treated by partial repeated complete metastasectomy, without ever having received systemic therapy compared to systemic therapy starting at first line with subsequent systemic therapy at tumor progression (11.3 vs. 3.1 years, HR 0.34, *p* = 0.002). Furthermore, also curative intended first-line complete metastasectomy and subsequent systemic therapy at tumor progression was associated with a significant survival benefit vs. systemic therapy starting at first line (5.8 vs. 3.1 years, HR 0.53, *p* = 0.003), even when an IO-based therapy was chosen in first line (5.8 vs. 3.5 years, HR 0.30, *p* = 0.017). However, for these first-line IO-based therapies, the significant difference in CSS vs. complete metastasectomy followed by systemic therapy for tumor progression was confirmed only compared to the IO–IO combination, but not compared to first-line TKI–IO combination (for IO–IO: HR 4.17, *p* = 0.013, for TKI–IO: HR 2.27, *p* = 0.283). In addition, median CSS appeared to be better in patients treated with partial repeated complete metastasectomy, without ever receiving systemic therapy, than in patients with first-line complete metastasectomy followed by systemic therapy at tumor progression, although this difference was not statistically significant (median CSS 11.30 vs. 5.77 years, *p* = 0.072).

Supplementally, median PFS, defined as time of first to second metastasis or tumor progression depending on first-line therapy, narrowly missed the significance level with IO-based therapy vs. TKI monotherapy (18.4 vs. 10.7 months, HR 0.61, *p* = 0.05), with maximal benefit of the TKI–IO combination vs. TKI monotherapy (38.0 vs. 10.7 months, HR 0.27, *p* = 0.01), as well as vs. curative complete metastasectomy (38.0 vs. 11.2 months, HR 0.34, *p* = 0.035). Median PFS with an IO–IO combination in first-line therapy was 8.9 months (range 0.4–33.6 months) with no significant difference compared to first-line therapies with a TKI monotherapy (HR 0.91, *p* = 0.740) or complete metastasectomy (HR 1.06, = 0.827). Figures 1C and 2A, B provide a summary of calculated PFS depending on first-line therapy, as well as univariate analyses for PFS and CSS.

## Discussion

The emergence of several new systemic therapeutic options is continuously changing the treatment paradigm in mRCC. Nevertheless, surgical resection of the primary tumor and metastatic lesions offers a definitive curative option in well-selected patients. Despite the lack of randomized controlled trials, there is evidence to support the benefit of metastasectomy in terms of CSS and overall survival (OS) in large observational studies during the cytokine era. For example, Sun et al. identified 1.976 patients in the National Cancer Database, treated with metastasectomy between 2006 and 2013, and demonstrated a 27% reduction in all-cause mortality through metastasectomy [9]. Another single-institution study of 97 patients who underwent metastasectomy between 2006 and 2017 showed significant improvement in OS and delay in time to initiation of targeted therapy for patients treated with complete metastasectomy [10]. In a systematic review, for the majority of studies, complete metastasectomy was favored with significantly longer survival rates compared with incomplete or no metastasectomy. All included studies, however, were non-randomized, comparative observational studies [8]. In a recent meta-analysis, Zaid et al. identified 8 retrospective studies involving more than 2.200 patients, of whom 42.3% received complete metastasectomy compared with 57.7% without or with incomplete metastasectomy between 1976 and 2013. Median OS ranged from 36.5 to 142.0 and 8.4 to 27.0 months for complete metastasectomy and no/incomplete metastasectomy, respectively. In addition, complete metastasectomy was associated with a reduced risk of all-cause mortality compared with incomplete metastasectomy (HR 2.37, 95% CI 2.03–2.87). As a limitation, only two of these studies included patients treated after 2008, whereas no study examined patients treated exclusively in the era of targeted therapy. This may bias the study results to overstate the oncologic efficacy of complete metastasectomy [6].

Our results are consistent with those of Li et al., showing an association of complete metastasectomy with significantly better OS (HR 0.5, 95% CI 0.25–0.98, *p* = 0.045). As a limitation, their cohort with targeted therapy only contained a significantly higher proportion of poor-risk MSKCC patients compared to the complete and incomplete resection groups (22.7 vs. 3.8 and 0%, respectively, *p* = 0.006). Therefore, the favorable characteristics of patients who underwent complete metastasectomy may have influenced the results, leading to the better survival outcomes [11].

In our study, we found that complete metastasectomy was generally associated with improved CSS compared with no metastasectomy in the era of targeted therapy and even the availability of IO-based therapy in univariate and multivariate analyses. Unfortunately, the recurrence rate after complete metastasectomy for mRCC is high. In our study, 83.8% of patients subsequently developed new metastases after first-line complete metastasectomy with a median PFS of 11.24 months. Nevertheless, in our analysis, patients with partially repeated complete metastasectomy without ever having received systemic therapy had the longest CSS with a median of 11.3 years. Moreover, even first-line complete metastasectomy with subsequent systemic therapy given at tumor progression still showed better CSS than targeted therapy from first line onward, even if an IO-based therapy was chosen in first line. It should be noted that these observed improvements in CSS may be due to patient selection with the most favorable disease biology. An interesting observation in our study, which also certainly needs further investigation in larger patient cohorts, is that the difference of a TKI–IO combination or a complete metastasectomy in the first line with in both cases initiation of systemic therapy at tumor progression showed no significant impact on CSS. Here, it should also be noted that in our analysis, patients after complete metastasectomy did not receive an IO-based therapy in the subsequent therapy line. Thus, it is reasonable to assume that first-line complete metastasectomy may be as good as first-line TKI–IO combination therapy for CSS, and perhaps the sequence of first-line complete metastasectomy followed by TKI–IO combination therapy for tumor progression may even achieve better CSS overall if repeat complete metastasectomy is not indicated.

Besides the fact of improved survival after complete metastasectomy, the fact of avoidance of toxicity by systemic therapy is also worth mentioning. In our study, the median time after complete metastasectomy until the next metastasis occurred was 11.24 months (range 0.36 months–11.6 years). This means just about 1 year without drug-related toxicity with potentially better quality of life avoiding chronic TKI toxicity or IO-induced side effects. However, it should be qualified that in our study the side effects and quality of life while on systemic therapy and after complete metastasectomy were not recorded, so this statement is hypothesis generating. Thus, complete metastasectomy followed by observation may have the potential advantage of sparing patients additional morbidity of systemic agents while preserving the efficacy of these agents for use later in the disease course. Actually, after complete metastasectomy, surveillance is recommended based on negative study results of adjuvant treatment with sorafenib or pazopanib [12, 13]. The recently published results of the KEYNOTE-564 trial are promising and lead to the approval of pembrolizumab for adjuvant treatment in RCC at high risk for recurrence or after complete metastasectomy in November 2021 by the FDA is recommended by guidelines [14]. In this study, adjuvant treatment with pembrolizumab significantly improved disease-free survival compared with placebo in the ITT population after surgery in patients with RCC who were at high risk for recurrence (disease-free survival at 24 months, 77.3% vs. 68.1%; HR for recurrence or death, 0.68; 95% CI, 0.53–0.87; *p* = 0.002). A subgroup of M1 no evidence of disease (NED) patients had the most prominent effect of adjuvant pembrolizumab compared to placebo (HR 0.29) [15].

Certainly, careful selection of patients for the therapeutic approach of complete metastasectomy is critical for success. Thus, solitary metastasis and prolonged disease-free interval between nephrectomy and metastasis are among known prognostic features of complete metastasectomy [16, 17]. Fittingly, our patients in the subgroup with first-line metastasectomy most frequently had metastasis in only one organ system (86.3%) and a median of only a single metastasis (range 1–8). However, the group was overall very heterogeneous with a wide variety of metastasis locations and sizes, so no reliable recommendation can be made as to which patients benefit from metastasectomy based on the available data. Potentially, new approaches, such as the analysis of genetic subtypes of RCC, may contribute to patient selection for or against metastasectomy, predicting with an increased likelihood in which patients are at higher risk of early recurrence after metastasectomy and would therefore benefit from alternative treatment options. In their study, Verbiest et al. identified four molecular subtypes of clear cell RCC (ccRCC1-4) and investigated their impact after metastasectomy. Here, the intermediate/poor prognostic ccRCC1 and ccRCC4 tumors had a significantly higher risk of recurrence and the good prognostic ccRCC2 and ccRCC3 subtypes had a longer disease-free survival [18].

Notable limitations of the study presented are the retrospective study design of our analysis and the lack of an assessment of functional or comorbid status after metastasectomy or while receiving systemic therapy. Moreover, the data on the distribution of number and size of metastases in the subgroup of patients with first-line systemic therapy, which are key factors for surgical approach, are not available and limit further statistical analysis. However, after reviewing the written radiologic reports, the metastatic burden exceeded the oligometastatic stage in the group of patients receiving systemic therapy. In addition, the number of patients with current guideline-recommended systemic therapies consisting of TKI–IO or IO–IO combinations in first line represents only a minority of patients in the study conducted. Finally, the results may not be generalizable because they represent the experience at a single institution.

In conclusion, despite the advances in targeted therapies and checkpoint inhibitors in first-line treatment of mRCC, our data suggest that complete metastasectomy remains an essential component of multimodality treatment of mRCC in the post-cytokine era. Therefore, complete metastasectomy may be considered in appropriately selected patients after a process of shared decision making.
